# Effects of energy spectrum on dose distribution calculations for high energy electron beams

**DOI:** 10.4103/0971-6203.48715

**Published:** 2009

**Authors:** Abdelkader Toutaoui, Nadia Khelassi-Toutaoui, Zakia Brahimi, Ahmed Chafik Chami

**Affiliations:** Département de Physique Médicale, Center de Recherche Nucléaire d'Alger; 2 Bd Frantz Fanon BP399 Alger RP, Algiers, Algeria; 1 Laboratoire de Sciences Nucléaires, Faculté de Physique, Université des Sciences et de la Technologie Houari Boumedienne, BP 32 El Alia, Bab Ezzouar, Algiers, Algeria

**Keywords:** Effective energy spectrum, electron pencil beam algorithm, monte carlo

## Abstract

In an early work we have demonstrated the possibility of using Monte Carlo generated pencil beams for 3D electron beam dose calculations. However, in this model the electron beam was considered as monoenergetic and the effects of the energy spectrum were taken into account by correction factors, derived from measuring central-axis depth dose curves. In the present model, the electron beam is considered as polyenergetic and the pencil beam distribution of a clinical electron beam, of a given nominal energy, is represented as a linear combination of Monte Carlo monoenergetic pencil beams. The coefficients of the linear combination describe the energy spectrum of the clinical electron beam, and are chosen to provide the best-fit between the calculated and measured central axis depth dose, in water. The energy spectrum is determined by the constrained least square method. The angular distribution of the clinical electron beam is determined by in-air penumbra measurements. The predictions of this algorithm agree very well with the measurements in the region near the surface, and the discrepancies between the measured and calculated dose distributions, behind 3D heterogeneities, are reduced to less than 10%. We have demonstrated a new algorithm for 3D electron beam dose calculations, which takes into account the energy spectra. Results indicate that the use of this algorithm leads to a better modeling of dose distributions downstream, from complex heterogeneities.

## Introduction

Radiation therapy with electron beams is now in widespread use because of the relatively uniform dose from the surface to a depth determined by the incident energy and the rapid dose falloff after this depth. Using these kinds of beams, it is possible to minimize irradiation of healthy tissues beyond the treatment volume. ‘Pencil’ beam models are still widely used in the field of radiotherapy for the calculation of dose distributions of clinically applied electron beams,[1–4] although in these last years, considerable work has been done to make the Monte Carlo calculation technique clinically available for electron beam calculations.[5–8] The majority of 3D treatment planning systems commercially available, employ pencil beam algorithms,[1,2,9,10] which assume that broad beams are composed of a finite number of pencil beams.

The most widespread approach used to determine pencil beam distribution has been to apply the Fermi-Eyges small angle multiple scattering theory.[1,2] However, it has been shown[11,12] that these type of algorithms may produce large errors (up 20%) when small, dense inhomogeneities are present in an otherwise homogeneous medium. Alternatively, the pencil beam distributions may be calculated using the Monte Carlo method,[2,13] and the idea to use precalculated pencil beam kernels has led to the development of the Monte Carlo-based pencil beam algorithm for treatment planning.[3,9,10,14,15]

An algorithm for 3D electron beam dose calculations based on Monte Carlo generated electron pencil beams was developed previously[13,15] in order to improve the accuracy of the small-angle multiple scattering theory-based pencil beam algorithm, In this model the electron beam is represented by a collection of monoenergetic pencil beams and the effects of the energy spectrum are taken into account by correction factors, derived from the measured central-axis depth dose curves.

In the present work, a model for electron dose calculations, based on Monte Carlo generated pencil beams, using an effective electron energy spectrum at the incident surface of the phantom, is described. The effective spectrum is determined from the depth-dose distributions measured in water.

Of particular importance is the determination of the incident energy distribution at the patient surface. In electron beams, electrons scattered from the collimator structure and beam modifiers produce low-energy electrons that are incident on the patient obliquely to the beam direction. The principal effect of these low-energy scattered electrons is more energy absorption than expected, at shallow depths. For an actual clinical beam, this effect is taken into account by finding a linear combination of monoenergetic electron kernels (essentially an incident electron energy spectrum) that account for the measured depth dose. In addition, the incident radiation beam on the patient requires a description of the beam penumbra. The penumbral effects are included by imposing a convolution on the beam pencil weights, with the spread parameter of the convolution filter function determined from measurements of the electron beam penumbra at different collimator-to-skin distances. This approach was suggested by Mohan *et al*.[16]

## Materials and Methods

### Dose calculation formalism

At present, most treatment planning calculations are based on the pencil-beam model,[1–4] which assumes that broad beams are composed of a finite number of pencil beams. This model is based on the Fermi-Eyges theory of the diffusion of electrons from a monodirectional point source, in the case where the linear angular scattering power *k* depends only on the depth *z* and not on the lateral position *r*. The present theory assumes that all electrons in the pencil beam lose the same amount of energy per unit depth, regardless of the lateral displacement and energy straggling that result from multiple Coulomb scattering (MCS). Electrons that are scattered away from the central axis of the pencil beam have traveled farther and therefore have lost more energy than electrons along the central axis. Because the limitation results from the approximation of the Fermi-Eyges theory, the theory is not used in pencil-beam models for clinical treatment planning without modification. In order to get more accurate results, a number of investigators have improved the Fermi-Eyges theory and proposed pencil-beam models.[1–4] These models constitute improvements over the original pencil-beam model resulting from the direct application of the Fermi-Eyges theory, while at the same time using this small-angle scattering theory as a starting point.

Alternatively, the pencil-beam dose distributions can be calculated from experimental measurements[17,18] or by the Monte Carlo method.[13]

In the present pencil beam model, the problem of dose calculation can be formulated as an integral equation, which expresses the resultant dose distribution in the patient for a given incoming electron beam:[19]

(1) D(r)=∫∫s∫∫∫h(E,Ω,r,ρ)·φE,Ω(ρ)·dE·dΩ·d2ρ

where *h* (E,Ω,*r*,ρ) is the energy deposition kernel representing the mean specific energy imparted at *r* per electron of energy E and direction Ω, incident at ρ, and φ_E_,Ω(ρ) is the incident particle fluence differential in energy and angle, at a point ρ on the patient surface. For h the most suitable unit is J·kg^−1^ and for φ it is m^−1^, thus the most suitable unit for φ_E_,Ω is m^−2^ MeV^−1^ sr^−1^. The spatial integrals have to be performed over the relevant entrance surface, S, of the patient.

If the energy and spatial distributions are not supposed to be correlated and can be modeled independently, the incident particle fluence differential in energy and angle becomes:

(2) $$\Phi_{E,\Omega }(\rho )=F_{E}(\rho )\cdot I(\rho )$$

where F_E_ is the incident electron beam energy spectrum and *I* is the relative intensity distribution of electrons on the surface of the patient. Moreover, it will be supposed that the energy spectrum does not depend on the lateral position (F_E_ (ρ) = F_E_).

Equation (1) can be then rewritten, in Cartesian coordinates, as follows:

(3) $$D(x,y,z)=\begin{array}{c} E_{\mathrm{Max}}\\ \int \\ 0 \end{array}F_{E}\mathrm{dE}\cdot \int \int I(x^{\prime },y^{\prime })\cdot h(E,x–x^{\prime },y–y^{\prime },z){\mathrm{dx}}^{\prime }{\mathrm{dy}}^{\prime }$$

E_max_ represents the maximum energy of the therapeutic electron beam energy spectrum. In its turn, the energy spectrum of the beam is discretized at regular intervals, hence the previous equation can then be written as:

(4) D(x,y,z)=Σi∫∫ΔEiFEdE·∫∫ I S(x′,y′)·h(E,x-x′,y-y′,z)dx′dy′=ΣiFi·∫∫SI(x′,y′)·h(Ei,x-x′,y-y′,z)dx′dy′

where F_j_ represents the j^th^ component energy spectrum of the broad beam corresponding to the energy E_j_.

### Monte carlo pencil beam algorithm

The core of the Monte Carlo pencil beam algorithm for electron dose calculation can be divided into two main parts: (i) the generation of the pencil beam kernel distributions according to the treatment geometry characteristics and effective energy spectrum, and (ii) the integration of the resulting kernel distributions over the entire beam aperture, to yield the dose. The algorithm uses a database of pencil beam kernels (a function of depth and radius only, due to the rotational symmetry of a pencil beam) that have been Monte Carlo calculated and stored for integer value energies between 1 and 50 MeV, in water.

To calculate the relative intensity distribution, the spatial spread resulting from the angular spread is convolved with a two-dimensional step function, wherein the shape corresponds to the applicator aperture. The angular spread is obtained by the deconvolution of measured intensity profiles as outlined by Mohan *et al*.[16]

The integration of the kernel distributions to yield a dose is performed following the scheme proposed by Ahnesjö and Saxner:[9] a circular region around the dose point is selected to be large enough to include the most widespread kernel possible at this depth. This region is further subdivided into smaller rings (all with their centers in the dose point), which in their turn are subdivided into segments. The dose contribution from electrons passing through a particular segment is determined by performing a ray trace through the patient to the center of the segment and calculating the spread of a pencil beam in this direction, resulting from interactions with the medium. This pencil beam is then integrated over the segment and the resulting dose is added to the dose at the dose point. The field shape is considered by projecting the field shape onto the integration plane and then integrating only over the part of the segment that lies inside the field. This is done by deriving a correction factor for each segment which measures the fraction of the segment that lies inside the field.

The effects of heterogeneities are considered by picking a kernel shape at an equivalent energy and depth in water as that computed for the heterogeneity with Gaussian theory and the infinite slab approximation.[20]

### Calculation of electron pencil beam kernels

In this study, EGS4-PRESTA-I Monte Carlo[21,22] was used to obtain energy deposition kernels from a monoenergetic electron pencil beam, with the incident energy between 1 and 50 MeV, interacting at the surface of a large cylindrical homogeneous water phantom. EGS4 is a Class II Monte Carlo code in which the effects of only a subset of the interactions for each type are grouped and treat the effects of the remaining interactions on an individual basis. In this class of schemes one excludes the grouping of individual collisions (denoted as catastrophic) in which energy losses or angular deflections are very important (greater than an arbitrary threshold). The history of an electron is divided into steps within which no “*catastrophic*” events happen. It is usually assumed that the noncatastrophic segment is described with reasonable accuracy by multiple collision models as the continuous slowing down approximation for energy losses and the multiple scattering theories for angular deviation.

Individual treatment is given to those relatively rare catastrophic interactions that create secondary particles above the same arbitrary energy. Discrete interactions cause the primary electrons to lose energy and get deflected. There are two catastrophic cut-offs for electrons, AE for secondary electron production (knock-on electrons) and AP for bremsstrahlung production. There are two other cut-offs, ECUT for electrons and PCUT for photons. These are the energy limits at which particle histories are terminated for electrons and photons, respectively.

For the EGS4-PRESTA-I code the electron transport is governed by the Molière multiple scattering theory, with the transport step size restricted so as not to allow for energy loss during a step to exceed a pre-set fraction (variable name ESTEPE in the EGS code) of the electron's energy. The continuous part of the energy loss is modeled using the *restricted stopping power*. This is the part of the stopping power restricted to creating secondary particles with energies less than the thresholds, AE and AP. For these simulations, the transport parameters AP = PCUT = 10 keV, AE = ECUT = 521 keV, and ESTEP = 2% were used. This value was selected in a conservative way, in order to eliminate any ESTEPE dependence of the simulations.

The PRESTA (Parameter Reduced Electron Step Transport Algorithm) version of EGS4[22] extends the original transport code. It consists mainly of three parts: the path-length correction (PLC) algorithm, which is based on the multiple scattering theory of Molière and takes into account the differences between the straight path length and the total curved path length for each electron step; a lateral correlation algorithm (LCA), which takes into account lateral transport; and a boundary crossing algorithm (BCA), which ensure that electrons are transported accurately in the vicinity of the interfaces.

**Figure 1:**
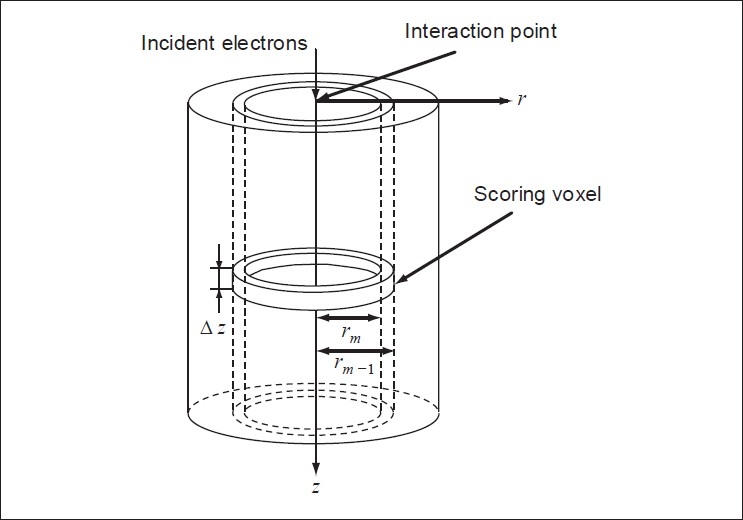
The geometry of the simulation phantom, with the interaction point and one ring-shaped scoring voxel indicated

An EGS4 user code called DOSRZ,[23] modeling the passage of an electron or photon beam in a finite, right cylindrical geometry, is used to simulate the energy deposition distributions induced by electron pencil beams, impinging normally on a homogeneous cylinder of water with a radius of 50cm and a height of 60cm. The water phantom is divided into scoring voxels. The voxel boundaries were defined at the intersection of 50 cylinders and 24 planar shells [Figure 1]. The user code DOSRZ generates pencil beam kernels in terms of an absorbed dose in each voxel, normalized to the total fluence of the incident primary electrons which interact at the origin. The pencil kernels are stored in tables of 50 and 24 radial and depth bins. The calculation of pencil beams using a coupled electron-photon Monte Carlo code has the advantages of estimating the electron scatter more accurately and including the dose from secondary particles. Therefore, the Monte Carlo pencil beam describes the energy deposition more accurately than the analytical pencil derived from the small-angle multiple scattering theories.

### Calculation of the effective energy spectrum

In the present Monte Carlo pencil beam algorithm, an effective energy spectrum is used to describe the incident energy at the patient surface element subtended by each pencil beam. The effective energy spectrum reproduces, with the highest possible degree of accuracy, the depth dose curve of clinical electron beams. The clinical beam is regarded as being a linear combination of the monoenergetic beams constituting this spectrum.

In the following, the numerical approach used for the effective energy spectrum of the electron beams on the surface of the phantom, using central axis depth-dose curves, will be presented.

Generally, the absorbed dose in any point of a medium irradiated by an electron beam of high energy is given by the dose integral equation (equation 3):

D(x,y,z)=∫0Emax⁡FEdE·∬SI(x′,y′)·h(E,x−x′,y−y′,z)dx′dy′

in cylindrical coordinates this equation becomes:

(5) $$D\left(x,y,z\right)=\int_{0}^{E_{\max}}F_{E}dE\cdot \iint_{S}I\left(x\prime ,y\prime \right)\cdot h\left(E,x-x\prime ,y-y\prime ,z\right)dx\prime dy\prime$$

The central axis depth-dose is obtained by integrating over the field size of the beam:

(6) $$D\left(z\right)=\int_{0}^{E_{0}}\int_{0}^{R}F_{E}\cdot I\left(r\prime \right)\cdot h\left(r\prime ,z,E\right)2\text{π}r\prime dr\prime dE$$

The irradiated medium is discretized in voxels (elementary volumes) defined by the intersection of concentric cylinders, around the central axis of the beam, and plans parallel to the surface of irradiated medium. *h*(*r, z, E*) remains constant inside a voxel. The central axis dose will then be written as:

(7) $$D\left(z\right)=\int_{0}^{E_{\max}}F_{E}dE\sum_{i}h_{i}\left(z,E\right)\int_{\Delta r_{i}}2\text{π}r\prime dr$$

(8) $$D\left(z\right)=\int_{0}^{E_{\max}}F_{E}dE\sum_{i}h_{i}\left(z,E\right)\text{π}\left(r_{i+1}^{2}-r_{i}^{2}\right)$$

The energy spectrum of the beam is discretized at regular intervals. Since the variation of *h_i_ (z,E)* is weak with the given energy bin, this equation can be discretized as follows:

(9) $$D\left(z\right)=\sum_{i}\left(\sum_{i}h_{i}\left(z,E_{i}\right)\text{π}\left(r_{i+1}^{2}-r_{i}^{2}\right)\int_{\Delta E_{i}}F_{E}dE\right)$$

DE_j_ is the jnth interval of the energy spectrum.

Now let us write this equation for each increment of depth z_k_:

(10) $$D\left(z_{k}\right)=\sum_{i}\left(\sum_{i}h_{i}\left(z_{k},E_{i}\right)\text{π}\left(r_{i+1}^{2}-r_{i}^{2}\right)\int_{\Delta E_{i}}F_{E}dE\right)$$

After the summation at all the radial distances we will have:

(11) $$d\left(z_{i},E_{i}\right)=\sum_{k}h_{k}\left(z_{i},E_{i}\right)\text{π}\left(r_{k+1}^{2}-r_{k}^{2}\right)$$

and

(12) $$\int_{\Delta E}F_{E}dE=\Phi_{i}$$

*d(z, E_j_)* represents the central axis depth-dose curve of a monoenergetic electron beam of E_j_ energy, and F represents the normalized effective energy spectrum of the broad beam.

By writing D_i_ = D(z_i_), equation (10) becomes:

(13) $$D_{i}=\sum_{i}d_{ij}\Phi_{i}$$

Provided the number of the depth at which the dose is measured is greater than or equal to the number of the energy bin, and *d*^1^ is not singular, equation (13) can be solved for F.

Since the central axis depth-dose of the broad beam can be measured and the central axis depth-dose of the monoenergetic beams can be determined by the Monte Carlo method, the resolution of the system gives the effective energy spectrum of the therapeutic beam.

The determination of the components of F involves constrained least-squares based on the minimization of the square differences called Non-*Negative Least Squares* method (NNLS).[24] This method ensures obtaining positive or null spectral components. The problem is sufficiently well-conditioned that the NNLS method can find a reasonable spectrum. In particular, the rapid fall-off of the measured depth dose as the range of the electron beam approaches will force to zero the coefficients (contributions) of any monoenergetic dose component of greater range. The uniqueness of the energy spectrum of a polyenergetic kernel is not critical in any case as long as the measurements of the depth dose are accurately reproduced. Monoenergetic depth dose curves are precalculated, for incident electron beam energies between 1 and 50 MeV, in steps of 1 MeV, using the EGS4-PRESTA Monte Carlo code.

## Results and Discussion

The effects of the inclusion of an initial energy spectrum with the Monte Carlo-based pencil beam algorithm for electron dose calculations in water, in presence of inhomogeneities, were studied by comparing calculations of this algorithm with measurements performed by Shortt *et al*.,[25] for the worst cases of small air and aluminium cylinders placed in a water phantom for a 10 MeV electron beam. These measurements were taken in a large water phantom containing cylinders of air and aluminium, simulating cavities of air and bone. The cylinder of air had a diameter of 1cm and a height of 2cm. The aluminium cylinder had a diameter and a height of 1cm. Measurements of depth dose curves and dose profiles were made for each cylinder, placed once at a depth of 2mm and another time at a depth of 2cm. These measurements were taken with a p-type silicon diode connected to an automatic data acquisition system. Overall uncertainty on measurements was lower than 2%.

Dose profiles and depth dose curves measured in the presence of air and aluminium cylinders were normalized to the dose at the depth of the maximum dose, for the open field (without inhomogeneities).

Calculations were carried out with the Monte Carlo pencil beam algorithm developed by convolving an effective spectrum with a polyenergetic pencil beam kernel. Another calculation was carried out with a monoenergetic pencil beam, where the effects of the energy spectrum were taken into account by correction factors, derived from the measured central-axis depth dose curves. Both Monte Carlo pencil beam algorithms used the same pencil beam kernel database calculated by the EGS4-PRESTA Monte Carlo code.

A variety of effective energy spectra, at the surface of the phantom, was first determined from the measured central axis depth dose by the NNLS method described above (equation 2). The calculated spectra comprised of a number of energy bins. The different energy spectra were obtained by varying the energy components (energy bins) of the effective spectrum.

Table 1 summarises the relative contribution to the effective spectrum of the different electron energy bins chosen.

**Table 1:** Proportion of monoenergetic energies included in various spectra determined from the NRCC 10 Mev electron beam

*Spectrum*	*Monoenergetic electron energy (MeV)*									
	
	*1*	*2*	*3*	*4*	*5*	*6*	*7*	*8*	*9*	*10*
1								0.229	0	0.852
2						0.155	0	0.02	0.035	0.905
3					0.124	0	0	0.101	0	0.9
4	0.053	0.035	0.023	0.010	0.064	0	0.052	0.065	0	0.904

**Figure 2:**
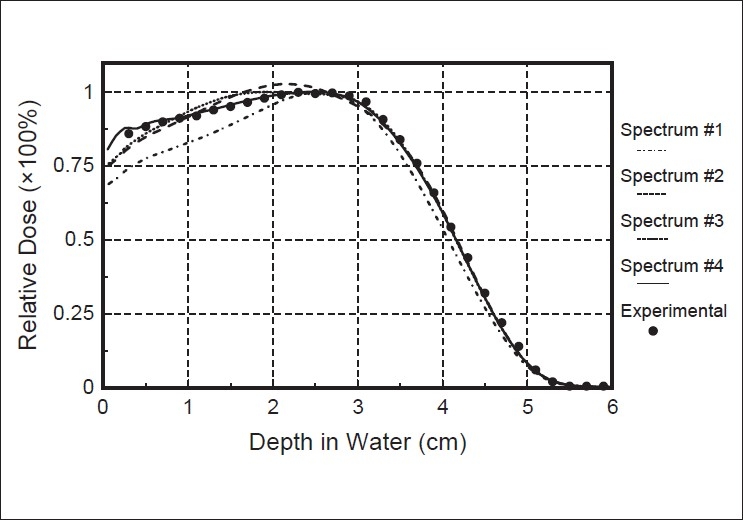
The effect of the effective spectrum on the central-axis depth dose curve. Comparison of reconstructed and measured depth-dose curves for a 10 MeV electron beam. The dashed and dotted line represents the depth-dose reconstructed from spectrum 1, the dashed line represents the depth-dose reconstructed from spectrum 2, the dotted line represents the depth-dose reconstructed from spectrum 3, and the solid line represents the depth-dose reconstructed from spectrum 4. Symbols represent the measured depth-dose curve

Figure 2 shows the central-axis depth dose curves reconstructed from these effective spectra. Spectrum 1 only includes contributions near the nominal accelerating energy. The depth dose curve from this spectrum is similar to the monoenergetic curve. Spectrum 4 gives the best agreement with the measured depth dose curve. Inclusion of energies less than 5 MeV improve the agreement near the surface.

The first comparison was done for dose distribution along the central axis for a 10 MeV electron beam in the case where low and high density cylindrical cavities were present in the water phantom.

**Figure 3:**
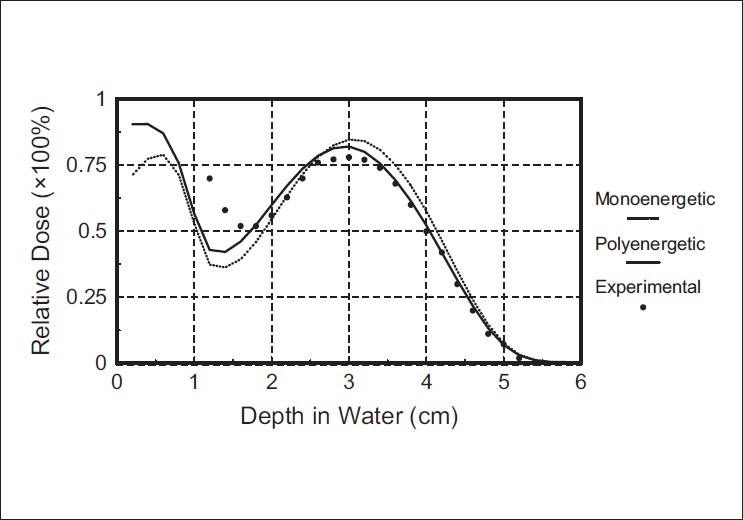
Comparison of central axis depth-dose for the aluminium cylinder placed at 2 mm, in water. Measured curve (full symbols), calculated curve by monoenergetic Monte Carlo pencil beam algorithm (dotted curve), and calculated curve by polyenergetic Monte Carlo pencil beam algorithm (solid curve)

**Figure 4:**
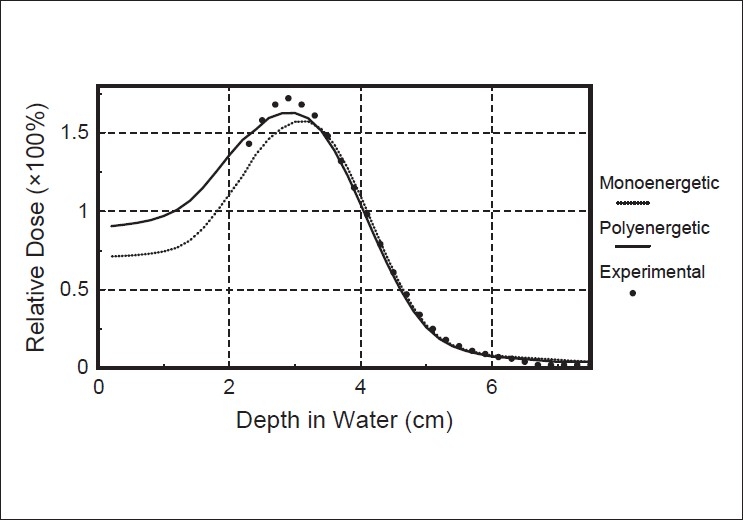
Comparison of central axis depth-dose for the air cylinder placed at 2 mm in water. Measured curve (full symbols), calculated curve by monoenergetic Monte Carlo pencil beam algorithm (dotted curve), and calculated curve by polyenergetic Monte Carlo pencil beam algorithm (solid curve)

Figure 3 shows the comparison of the calculated depth-dose curves by the polyenergetic pencil beam kernel algorithm and the monoenergetic pencil beam algorithm, against the measured depth-dose curve in the presence of a 1cm diameter, 1cm long aluminium cylinder. The cylinder's axis is parallel to the beam axis and the top of the cylinder is recessed from the water surface by 2mm.Figure 4 illustrates the same comparison for a 1cm diameter, 2cm long air cylinder placed at 2mm depth.

These figures illustrate the improvement resulting from the use of polyenergetic pencil beam kernels, in the prediction of the depth dose behind the inhomogeneity, compared with the use of monoenergetic electron pencil beams. The algorithm based on monoenergetic kernels predicts the cold spot beneath the aluminium cylinder with an error of 14%, whereas, the depth dose peak is about 8% larger than the experimental one. The use of polyenergetic kernels reduces this error to about 10% for the cold spot and less than 4% for the peak [Figure 3].

In the case of the air cylinder, the monoenergetic pencil beam algorithm predicts the hot spot with an error of about 20%, while the polyenergetic algorithm reduces this error to less than 10% [Figure 4]. This improvement is due to the fact that the low energy tail, despite its small value, has a considerable influence on the dose near the surface.

**Figure 5:**
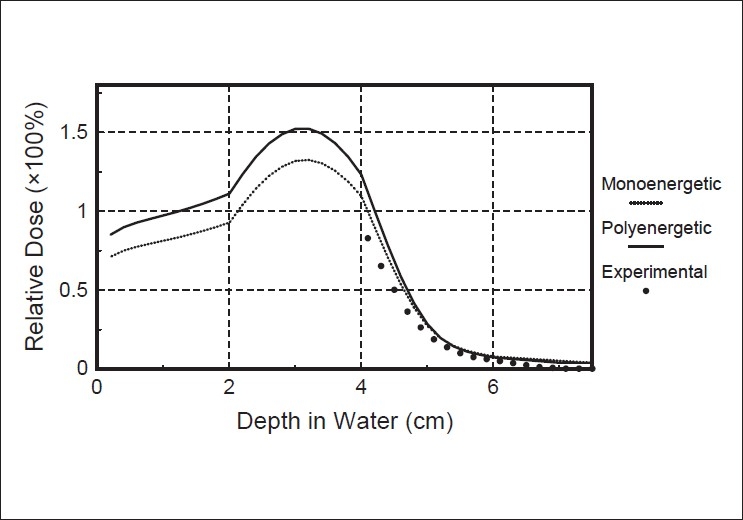
Comparison of central axis depth-dose for the air cylinder placed at 2 cm in water. Measured curve (full symbols), calculated curve by monoenergetic Monte Carlo pencil beam algorithm (dotted curve), and calculated curve by polyenergetic Monte Carlo pencil beam algorithm (solid curve)

Comparison between measured and calculated depth dose data for a 1cm diameter, 2cm long cylindrical air cavity placed at 2cm in water is given in Figure 5. It can be seen from these results that the two calculation models give the depth dose behind the inhomogeneity with the same magnitude of error. The predictions of the monoenergetic electron pencil model are approximately the same as those of the polyenergetic pencil beam algorithm, because the influence of the low-energy components in the effective spectrum, included in the polyenergetic pencil beam algorithm, is not very important at great depths, due to the limited range of low-energy electrons in water.

Following this, we undertook the comparison of calculated and measured dose profiles behind the inhomogeneous cavity. These comparisons were made for two different depths; one was located just below the cylinder and the other a little deeper.

**Figure 6:**
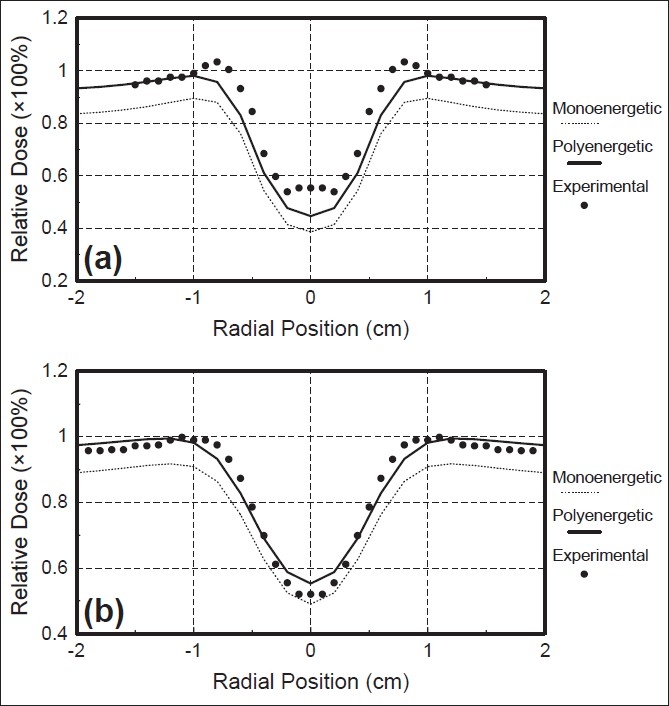
Radial dose profile for 10 MeV electron beam incident on the standard aluminium cylinder placed at 2 mm depth. (a) dose profile at 0.35 cm behind the cylinder, (b) dose profile at 1.15 cm behind the cylinder. Measured curve (full symbols), calculated curve by monoenergetic Monte Carlo pencil beam algorithm (dotted curve) and calculated curve by polyenergetic Monte Carlo pencil beam algorithm (solid curve)

The central parts of the radial dose profiles for a 10 MeV electron beam, calculated by the two algorithms and measured, behind the aluminium cylinder placed at 2mm, are compared and shown in Figure 6. The profiles were computed at 0.35 and 1.15cm, respectively, at the bottom of the inhomogeneity. It was seen that both algorithms could predict the sharp drop in the absorbed dose behind a small aluminium cavity. It was also noticeable that the agreement between the measured and calculated doses was better in the polyenergetic pencil beam algorithm. Just behind, the inhomogeneity [Figure 6a], the discrepancy between the calculation and measurement of the central axis of the profile was about 10% for the polyenergetic pencil beam algorithm, whereas, it was more than 15% for the monoenergetic pencil beam algorithm. Far from the central axis, the polyenergetic pencil beam algorithm predicted dose values with an error lower than 1%, while the calculated values by the algorithm were lower by 2% compared to the measured values.

At the second depth [Figure 6b], the agreement of the computed dose values by the two algorithms, with the measurements, was better. The difference between the measured value of the cold point at the central axis and those calculated by the two algorithms does not exceed 3%, whereas, the error on the off-axis calculated dose is not more than 5% for the monoenergetic pencil beam algorithm.

**Figure 7:**
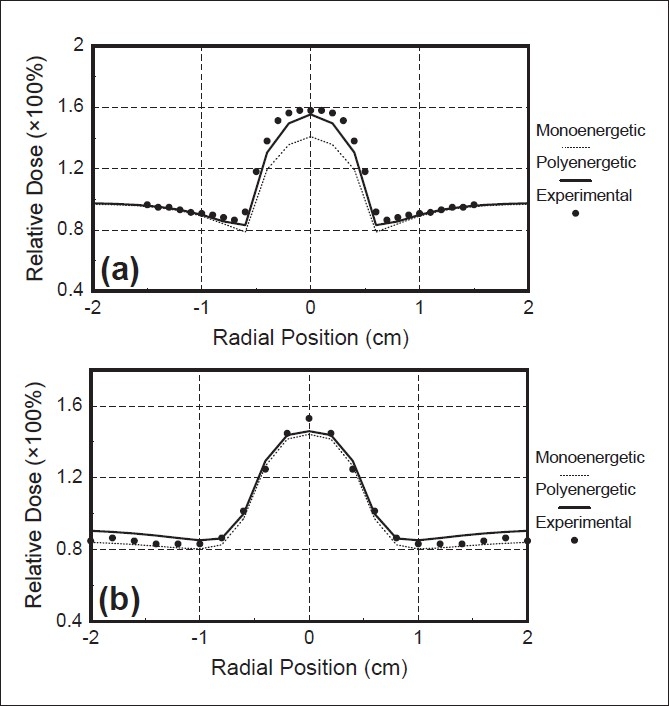
Radial dose profile for 10 MeV electron beam incident on the standard air cylinder placed at 2 mm depth. (a) dose profile at 0.35 cm behind the cylinder, (b) dose profile at 0.75 cm behind the cylinder. Measured curve (full symbols), calculated curve by monoenergetic Monte Carlo pencil beam algorithm (dotted curve) and calculated curve by polyenergetic Monte Carlo pencil beam algorithm (solid curve)

Figure 7 illustrates the comparison of the calculated and measured dose profiles for a 10 MeV electron beam, behind the air cylinder, placed at 2cm. The profiles were computed at 0.35 and 0.75cm, respectively, at the bottom of the air cavity. Both the Monte Carlo pencil beam algorithms successfully predicted the dose increase immediately behind the small air cavity, although the polyenergetic pencil beam algorithm results showed a much better agreement with the measurement, and accurately predicted the sharp dose increase behind the small air cavity [Figure 7a]. The results of both algorithms were comparable for the second depth. The hot point was predicted with an error of 7% [Figure 7b].

It can be seen that the polyenergetic algorithm improves the agreement between measured and calculated hot and cold spots just inside and outside the inhomogeneity, and at distances far from the cavities.

## Conclusion

Measured dose distributions have been compared with those calculated by the monoenergetic pencil beam and polyenergetic Monte Carlo based pencil beam algorithms for the same incident beam and phantoms. This study has demonstrated that the use of an effective energy spectrum derived from the measured central-axis depth dose curves, in water, to drive Monte Carlo polyenergetic pencil beams, leads to an improvement in the accuracy of electron beam dose calculations.

Better agreement was found between polyenergetic pencil beam calculations and the measurements in both depth-dose curves and dose profiles for the water phantom, in the presence of high- and low-density, small cylindrical cavities. The polyenergetic pencil beam can predict more accurately than the monoenergetic pencil beam, and hot and cold spots are caused by simple 3D inhomogeneities.

It has been shown that in inhomogeneous phantoms, both Monte Carlo-based pencil beam algorithms are able to predict the depth-dose distribution far from the inhomogeneity.

The comparison has demonstrated some serious limitations of the monoenergetic pencil beam algorithm to accurately predict hot and cold spots just behind the inhomogeneity. The polyenergetic pencil beam results generally have much better agreement with the measurements, especially in predicting sharp increases or decreases in the absorbed dose, caused by the perturbation of adjacent 3D inhomogeneities.

Results indicate that the use of this algorithm leads to a better modeling of dose distributions downstream from complex heterogeneities.
